# Multi-Mixed Metal Hydroxide as a Strong Stratigraphic Nanoclay Inhibitor in Solid-Free Drilling Fluid

**DOI:** 10.3390/nano12213863

**Published:** 2022-11-01

**Authors:** Bowen Zhang, Qingchen Wang, Weichao Du, Yongfei Li, Jianqing Zhang, Jie Zhang, Marián Matejdes, Michal Slaný, Chen Gang

**Affiliations:** 1 State Key Laboratory of Petroleum Pollution Control, Xi’an Shiyou University, Xi’an 710065, China; 2 Shaanxi Province Key Laboratory of Environmental Pollution Control and Reservoir Protection Technology of Oilfields, Xi’an Shiyou University, Xi’an 710065, China; 3 CNPC Chuanqing Drilling Engineering Company Ltd., Xi’an 710018, China; 4 Institute of Inorganic Chemistry, Slovak Academy of Sciences, Dúbravská Cesta 9, 845 36 Bratislava, Slovakia; 5 Institute of Construction and Architecture, Slovak Academy of Sciences, Dúbravská Cesta 9, 845 03 Bratislava, Slovakia; 6 Xi’an Key Laboratory of Tight Oil (Shale Oil) Development, Xi’an Shiyou University, Xi’an 710065, China

**Keywords:** multi-mixed metal hydroxide, bentonite, inhibitors, drilling fluid

## Abstract

Solid-free drilling fluid has more advantages as a new type of drilling fluid compared with traditional drilling fluid, such as improving drilling efficiency, protecting oil and not having clay particles clog the oil and gas layer. In this study, Zn/Cu/Fe-doped magnesium–aluminum hydroxide (Mg-Al MMH) was prepared using the co-precipitation method and evaluated in solid-free drilling fluid. The inhibition mechanism of synthesized hydroxide was analyzed by X-ray diffraction, laser particle-size analysis and thermogravimetric analysis. The samples were directly used as drilling fluid base muds for performance evaluation. The results showed that the linear expansion rate of 4% M6-Fe was only 12.32% at room temperature within 2 h, that the linear expansion rate was 20.28% at 90 °C and that the anti-swelling rate was 81.16% at room temperature, indicating that it has a strong inhibition ability at both room temperature and at high temperatures. Meanwhile, the possibility of multi-mixed metal hydroxide as a drilling fluid base mud is discussed in this study. We found that 4% M6-Fe exhibited low viscosity, a high YP/PV ratio and high temperature resistance, and its apparent viscosity retention rate reached 100% rolled at 200 °C for 16 h, with a YP/PV ratio of 2.33.

## 1. Introduction

Solid-free drilling fluid is a new type of drilling fluid that has more advantages than solid-phase drilling fluid. It can increase speed to shorten production cycles. Its low viscosity is conducive to high-pressure injection. The stable performance of solid-free drilling fluid is easy to maintain and handle and reduces the cost of drilling fluid [1]. A solid-free system has been used in specific low-pressure and low-permeability formations since the 1980s and has been applied to some extent [2,3]. A simple solid-free system was used in low-permeability, low-pressure formations, which contained only polymers and salts, and had a drilling fluid density of 1–1.75 g/cm^3^. However, temperature resistance is poor with a temperature no higher than 100 °C [4,5,6]. In the early 1990s, Block et al. developed a solid-phase drilling fluid with improved temperature resistance to 120 °C and better stability to metal ions in the tested formation [7]. The system is a dispersive colloid under a positive electric environment, which has a strong ability to inhibit clay dispersion and can protect oil and gas formation well. It has been used in the drilling process for several thousand wells in Chinese oil and gas fields, such as the Shengli Oilfield and the Dagang Oilfield. It has achieved impressive results [8,9,10]. In 1942, Feitknecht [11] stated that the structures of these compounds had double layers: a regular triakis octahedral layer containing divalent cations in another regular dioctahedral layer containing trivalent cations. The composition of chemicals can generally be described as follows:

[(M^II^)_1−*x*_(M^III^)*_x_*(OH)_2_]*^x^*^+^(A*^m^*^−^*_x_*_/*m*_)·*n*H_2_O] (1)

The cation M^2+^ in its chemical formula is bivalent, such as Mg^2+^, Mn^2+^, Fe^2+^, Co^2+^, Zn^2+^, etc. Moreover, M^3+^ is a trivalent metal cation, such as Al^3+^, Cr^3+^, Mn^3+^, Fe^3+^, Co^3+^, Ni^3+^ and so on. A^n−^ is an anion with a valence of n, such as Cl^−^, NO_3_^−^, etc. [12,13,14]. In 1988, Burba et al. [15] first proposed the use of a layered mixed metal hydroxide (MMH) in oilfield drilling. MMH products widely used in Chinese oil fields are typically magnesium–aluminum hydroxide (Mg-Al MMH) and contain Mg^2+^, Al^3+^, OH^−^ and Cl^−^ [16]. However, the study of multivariate mixed metal hydroxide (M-MMH) precipitation is not perfect. The current study is only for Mg-Al metals, and the effects of raw materials on the inhibition and rheology of the samples are not perfect. The study of the inhibition of M-MMH samples at high temperatures is also not perfect [17]. In this study, samples of M-MMH were synthesized by a co-precipitation method, using MgCl_2_, AlCl_3_, FeCl_3_, CuCl_2_ and ZnCl_2_ as raw materials. These materials are easily available and can be used in industrial applications. The inhibition performances of the synthesized samples at room temperature and at a high temperature were evaluated by a linear expansion experiment, an anti-swelling experiment and a mud ball experiment to determine the products with the most effective inhibition performances and their optimal concentrations in the experiment. The mechanism of the inhibition performance of synthesized samples was analyzed by X-ray diffraction experiments, SEM laser particle-size experiments and thermogravimetric experiments. Additionally, there are no studies reporting that samples can be directly used as a drilling fluid base mud. In this study, the possibility of the samples being directly used as a drilling fluid base mud was evaluated by drilling fluid performance tests. Therefore, this study is interesting and can contribute to the scientific community.

## 2. Experimental

### 2.1. Materials

Sodium bentonite (technical grade; mineralogical composition: 70% montmorillonite, 20% quartz, 10% feldspar) and calcium bentonite (technical grade; mineralogical composition: 60% montmorillonite, 25% quartz, 15% feldspar) were purchased from Xi’an Chanqing Chemical Co., Ltd., Xi’an, China. MgCl_2_, AlCl_3_ and NaOH (technical grade) were purchased from Tianjin Shengao Chemical Reagent Co., Ltd., Tianjin, China. FeCl_3_, CuCl_2_ and ZnCl_2_ (technical grade) were purchased from Xi’an Fengyun Chemical Co., Ltd., Xi’an, China.

### 2.2. Preparation of M-MMH Samples

In the experiment, different ratios of MgCl_2_, AlCl_3_, FeCl_3_, CuCl_2_ and ZnCl_2_ were put in 400 mL of water at room temperature. The specific additions and names are shown in Table 1. Eventually, precipitate was wholly seen in the beaker. The obtained mixed precipitate was sealed and aged at room temperature for 1 h [18,19]. The aged precipitate was centrifuged at 1500 r for 10 min to obtain the product.

### 2.3. Inhibitory Evaluation

The hydrated expansion of sodium bentonite was determined using a shale expander (NP01, Chuangmeng Ltd., Qingdao, China), according to the Chinese Petroleum and Natural Gas Industry Standards SY/T59711994 and SY/T63351997 [20]. Using a 2:1 mass ratio, we dried the sodium bentonite at 105 °C for two hours, then mixed it with water to form mud balls with a mass of about 10 g each. We then immersed the mud balls (sodium bentonite) in the same volume of different M-MMH treatment agents and took photographs after a certain period of time to observe shape changes, then evaluated M-MMH inhibition by the apparent changes and surface changes of the mud balls [21]. The effects of the inhibitor on the anti-swelling rate and shrinkage rate of bentonite were investigated based on SY/T5971-1994 [22].

### 2.4. Drilling Fluid Evaluation

M-MMH samples (4%) were added to tap water (350 mL), stirred for 30 min and aged for 16 h at 298 K. The rheological properties, filtration properties and lubrication properties of the drilling fluid, such as AV (apparent viscosity), PV (plastic viscosity) and YP (yield point), were evaluated using a viscometer (ZNN-D6S, Hetongda Co., Ltd., Qingdao, China), a medium-pressure filtration instrument (GJSS-B12K, Haitongda Co., Ltd., Qingdao, China) and a viscosity coefficient instrument (Qingdao Hetongda Co., Ltd., Qingdao, China), according to the formulas in the Chinese National Standard GB/T 16783.1-2006 [23].

### 2.5. Particle Size Experiment

The particle size of each sample was measured with a laser particle sizer to determine the median and mean particle diameters of each sample, and the data were used to analyze the variation in sodium bentonite particle size, which was measured with an Laser Particle Sizer instrument (LS-13320, Beckman Coulter, Inc., CA, USA) [24].

### 2.6. X-ray Diffraction Analysis

The samples were analyzed with a X-ray diffractometer (D8ADVANCE, Bruker, Inc., Saarbrücken, Germany). A Cu target, ceramic X-ray tube, tube current of 40 mA, tube voltage of 40 kV, step size of 0.02° and scanning range of 5~60° (2θ) were used for the measurements. The variation in the interlayer spacing of sodium bentonite under different conditions was calculated using the Bragg equation (nλ = 2dsinθ) [25].

### 2.7. TGA

Sodium bentonite was dispersed in M-MMH suspensions for 24 h. Bentonite was separated and dried at 378 K for TGA. TGA was performed with a thermal analysis machine (TGA/DSC1/1600, Mettler Toledo Inc., Zurich, Switzerland) [26].

### 2.8. SEM and TEM

The surface morphology of samples was investigated using a digital imaging scanning electron microscope (model SU6600, serial no. HI-2102-0003, Hitachi, Tokyo, Japan). The sample was subjected to an HRTEM analysis with a transmission electron microscope (Talos F200X, FEI Inc., OR, America) Cs probe microscope after sputtering of gold by ion sputtering for 45 s to explore the structural properties of the as-synthesized materials [27].

## 3. Results and Discussion

### 3.1. Linear Expansion Experiment

Bentonite is a kind of clay rock, also called montmorillonite rock, which contains small amounts of illite, kaolinite, quartz, feldspar, etc. Bentonite has strong hygroscopicity and expansion. It can adsorb 8 to 15 times its own volume of water, and volume expansion can increase 30 times. Therefore, bentonite was selected for this study [28]. Linear expansion experiments were carried out on all M-MMH samples. Each test was repeated at least three times to obtain average values. Meanwhile, the experiments were conducted in water with 4% KCl solution as a control, because KCl is widely used in industrial drilling fluid as a drilling fluid inhibitor. Based on the results obtained, the linear expansion rate of bentonite in water was 63.11%, and that in the 4.0% KCl solution was 46.17% within 2 h. 

**Figure 1:**
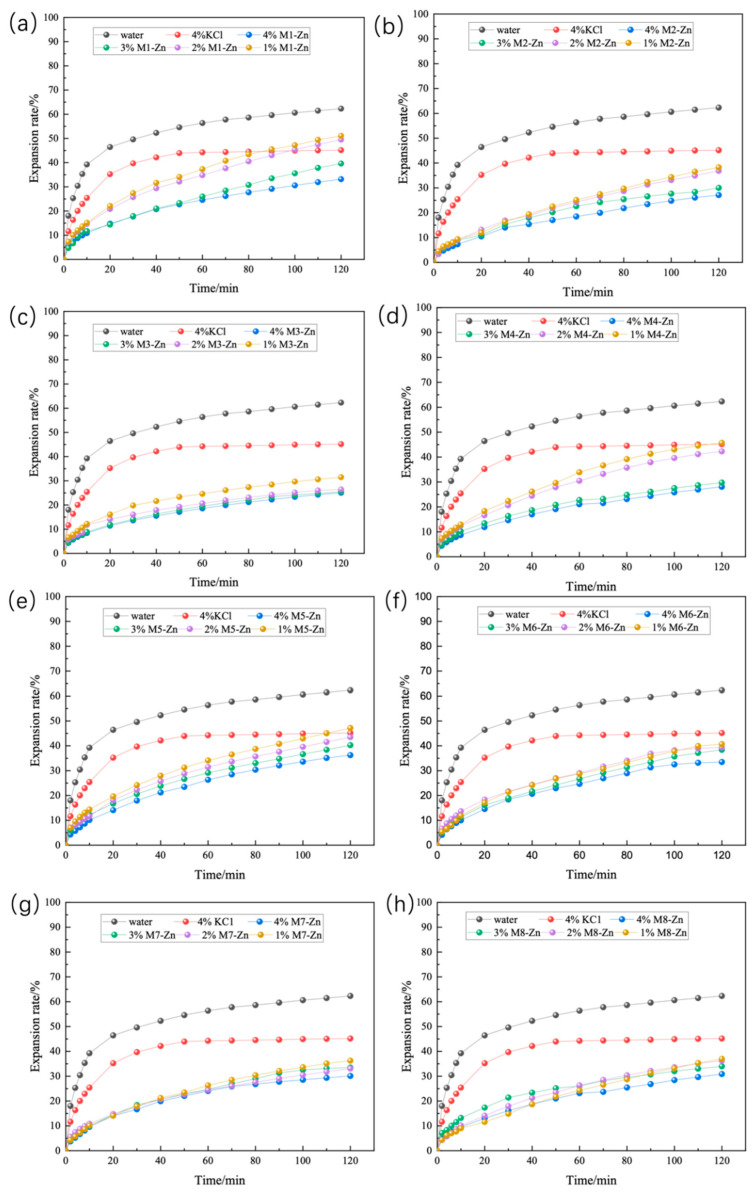
Linear expansion of Zn-doped MMH with different concentrations ((**a**): M1-Zn; (**b**): M2-Zn; (**c**): M3-Zn; (**d**): M4-Zn; (**e**): M5-Zn; (**f**): M6-Zn; (**g**): M7-Zn; (**h**): M8-Zn).

**Figure 2:**
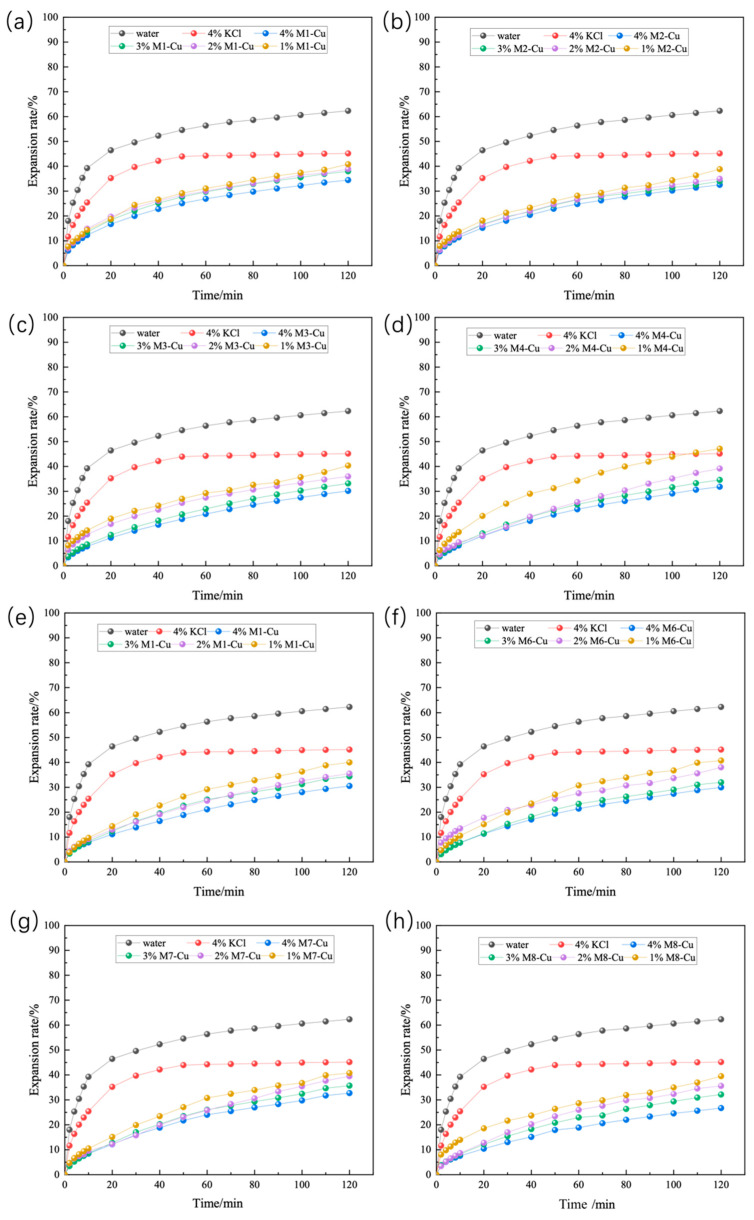
Linear expansion of Cu-doped MMH with different concentrations ((**a**): M1-Cu; (**b**): M2-Cu; (**c**): M3-Cu; (**d**): M4-Cu; (**e**): M5-Cu; (**f**): M6-Cu; (**g**): M7-Cu; (**h**): M8-Cu).

**Figure 3:**
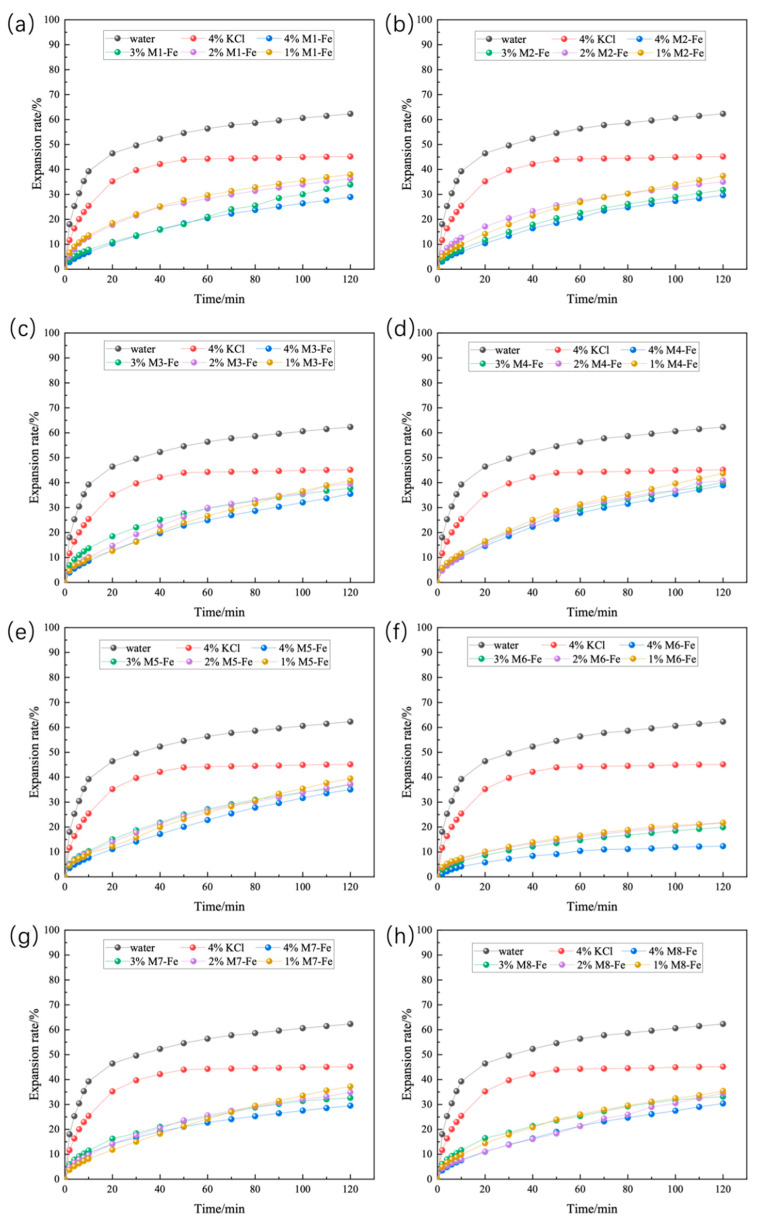
Linear expansion of Fe-doped MMH with different concentrations ((**a**): M1-Fe; (**b**): M2-Fe; (**c**): M3-Fe; (**d**): M4-Fe; (**e**): M5-Fe; (**f**): M6-Fe; (**g**): M7-Fe; (**h**): M8-Fe).

The results of linear expansion experiments on Zn-doped MMH samples with different concentration compacts at room temperature are shown in Figure 1. From Figure 1, it can be seen that inhibition increased with the increase in concentration and that M3-Zn had a more effective inhibition than the other Zn-doped MMH samples. The 4% concentration of M3-Zn showed a strong inhibition of hydration expansion, with an increase of 59.80% compared to water and of 44.53% compared to the 4% KCl solution. As can be seen from Figure 2, the performance of the Cu-doped MMH was similar to that of the Zn-doped MMH. Compared with water, M8-Cu increased 57.12%, and increased 40.83% compared to the 4% KCl solution. It can be seen from Figure 3 that the best-inhibited sample was M6-Fe. The linear expansion rate was 12.32% at room temperature and pressure, with an increase of 80.23% compared to water and of 72.72% compared to the 4% KCl solution.

**Figure 4:**
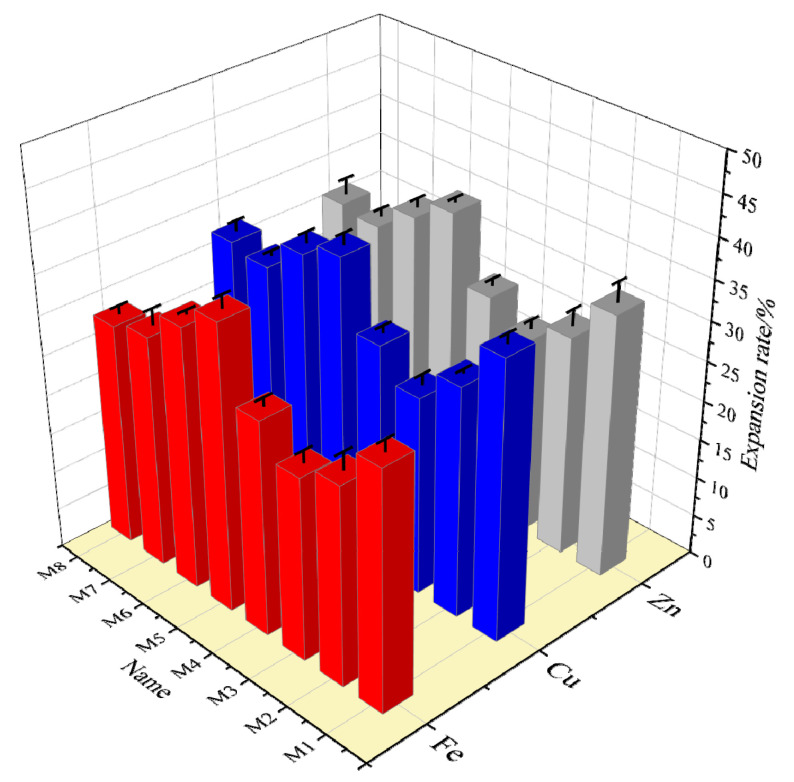
Graph of the linear expansion of different samples at 4% concentration at room temperature and pressure.

From the linear expansion experiments, it is known that the most effective inhibitory concentration is 4%. The results of the linear expansion experiments for all 4% samples are compared in Figure 4. From Figure 4, it can be seen that the sample with the best effect was M6-Fe and that its linear expansion rate for 120 min was only 12.32%.

### 3.2. High-Temperature Resistance Experiment

**Figure 5:**
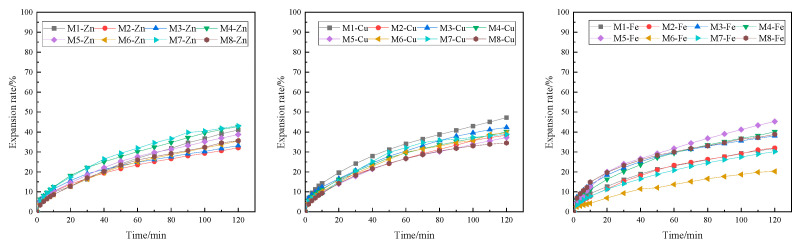
Curves of the linear expansion for the 4% concentrations of samples at 90 °C.

Based on Section 3.1, it is known that all the samples performed best at a concentration of 4%; consequently, the concentration of 4% was chosen in this study. From Figure 5, the linear expansion rate of clear water was 69.82% and that of 4% KCl was 46.12% at 90 °C. The samples still maintained excellent performance under high-temperature conditions and were overall better than the 4% KCl solution, of which M6-Fe had the best effect under room-temperature conditions and was still optimal at a high temperature. The linear expansion rate of 4% M6-Fe at 90 °C was 20.28%, with an increase of 70.95% compared to water and of 56.02% compared to the 4% KCl solution.

### 3.3. Anti-Swelling Experiment

**Figure 6:**
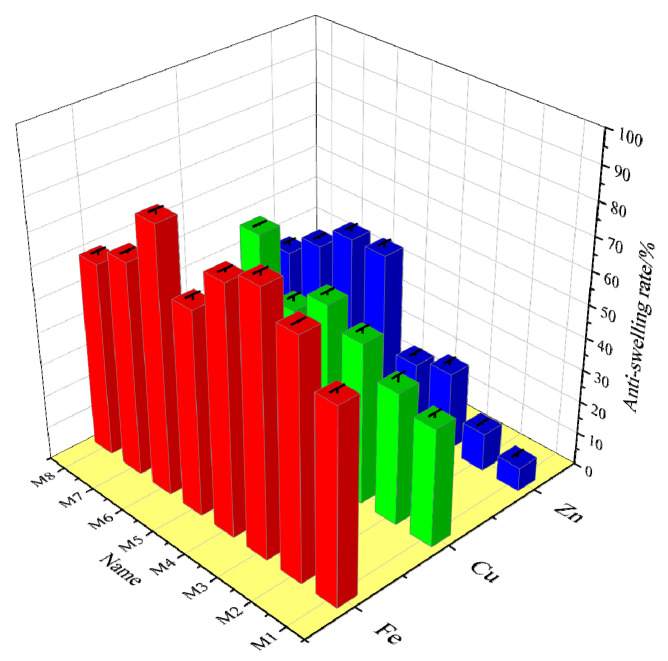
Anti-swelling rates for the 4% concentrations of different samples.

Referring to the experimental methods in Section 2.4, the effect of M-MMH samples on the anti-swelling rate of bentonite was investigated. Bentonite was fully expanded in water and then centrifuged so that the water between the pores of the bentonite was fully discharged to evaluate the anti-swelling effect. Figure 6 shows the anti-swelling rate of 24 samples at room temperature. M6-Fe showed the best inhibition, with an 81.16% anti-swelling rate, while Zn-doped MMH showed the worst overall anti-swelling effect; the worst inhibition was that of M1-Zn, with a 7.25% anti-swelling rate.

### 3.4. Mud Ball Experiment

**Figure 7:**
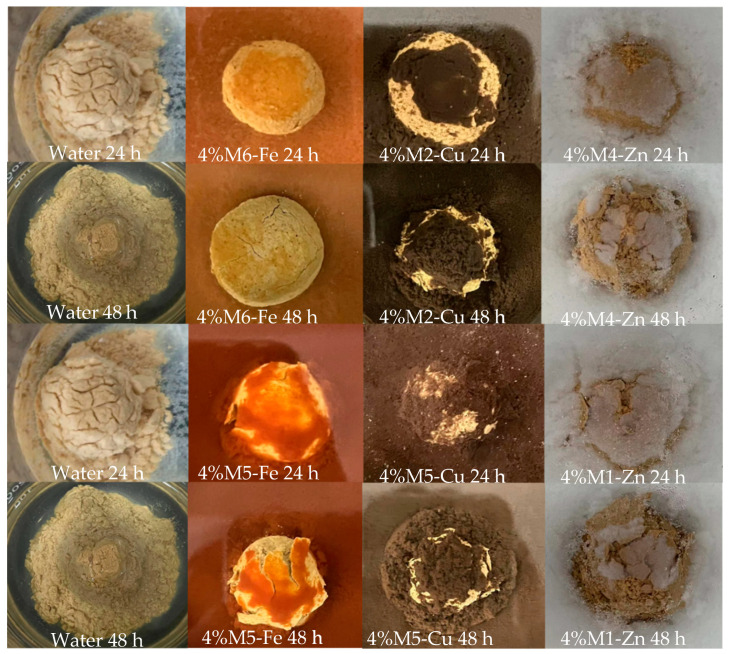
Experimental results for the mud balls.

The six samples (M1-Zn, M4-Zn, M2-Cu, M5-Cu, M5-Fe and M5-Fe) were selected by linear expansion experiments and anti-swelling experiments for use in mud ball experiments. It can be seen from Figure 7 that the six samples inhibited the hydration dispersion and hydration expansion of mud balls compared with water. The mud ball in water completely collapsed and dispersed after 48 h. The mud balls in the six samples of M-MMH kept their shapes intact. M6-Fe had the best inhibitory effect, and the mud balls remained intact after 48 h, with only some cracks appearing. The mud ball experiment proves that M-MMH samples can inhibit the hydration dispersion and hydration expansion of bentonite.

### 3.5. X-ray Diffraction Analysis

**Figure 8:**
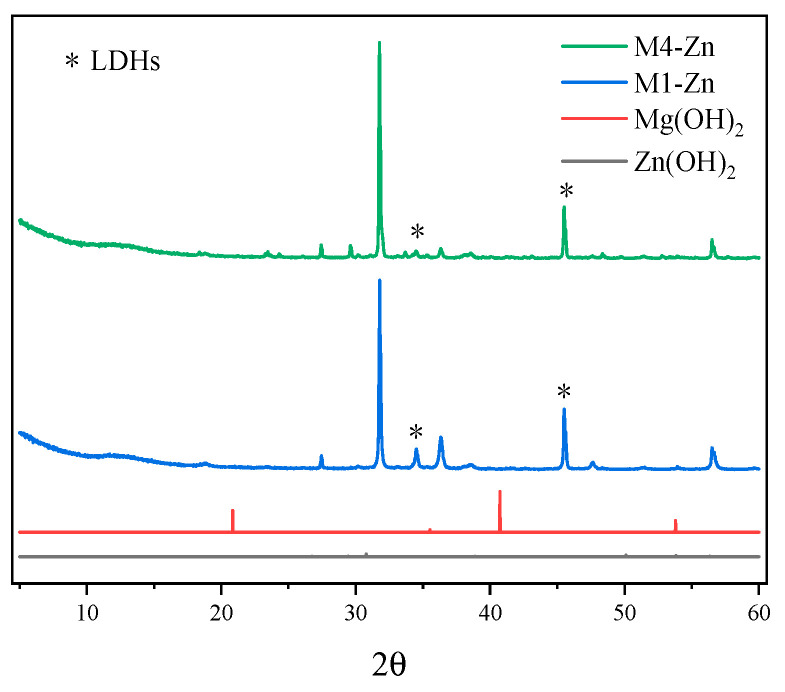
X-ray diffraction results for some Zn-doped MMH samples.

**Figure 9:**
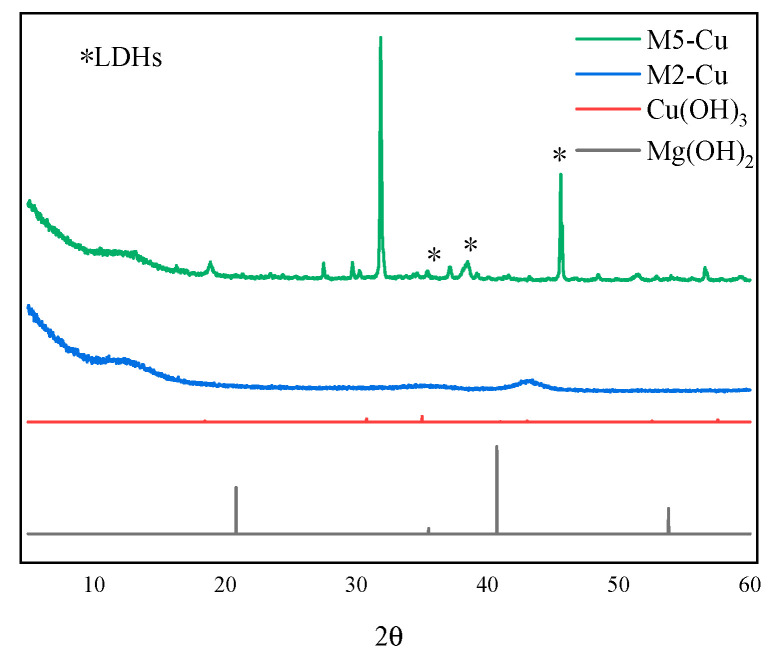
X-ray diffraction results for some Cu-doped MMH samples.

**Figure 10:**
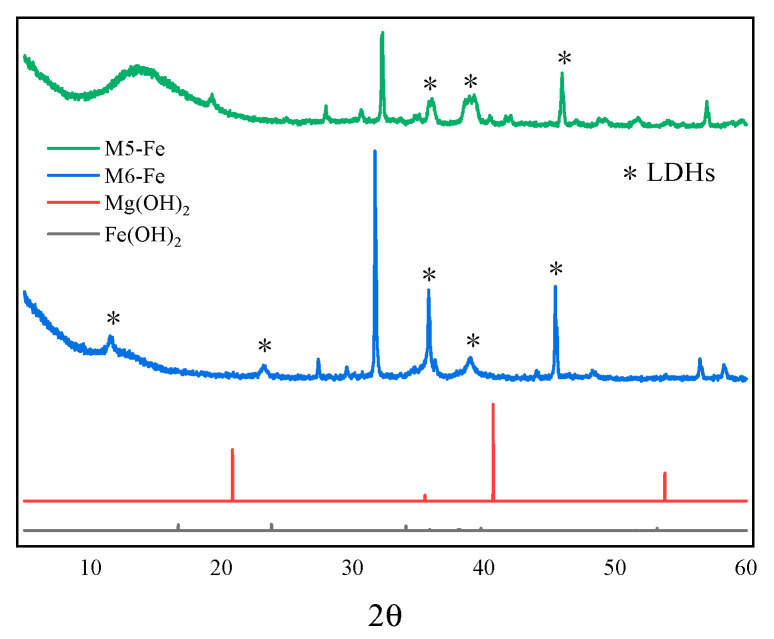
X-ray diffraction results for some Fe-doped MMH samples.

The results of the X-ray diffraction experiments are shown in Figure 8, Figure 9 and Figure 10 for some of the M-MMH samples with their corresponding metal ion hydroxide precipitates.

It can be seen from the figures that the characteristic peaks of the samples are different from those of Mg(OH)_2_ and also from those of the corresponding hydroxide precipitates of Zn, Cu and Fe, i.e., Zn(OH)_2_, Cu(OH)_2_ and Fe(OH)_3_. Thus, it can be seen that the synthesized sample of M-MMH is not a hydroxide precipitate corresponding to metal ions but a new substance. It has been proven by numerous experiments that M-MMH has a crystal structure, such as hydrotalcite [29]. M6-Fe has the most characteristic peaks of LDHs, which proves that it has the most complete crystal morphology.

### 3.6. Particle Size Measurement

Table 2 shows the particle size measurement of bentonite before and after the addition of M-MMH samples.

From Table 2, it can be seen that in clear water the average particle size of hydrated bentonite particles decreased from 36.42 μm to 17.12 μm, while the median particle size of bentonite particles increased after adding some 4% concentration samples to the hydrated bentonite base mud. The average particle size of bentonite increased from 17.12 μm to 38.75 μm after the addition of the M6-Fe sample. The results of the particle size measurement verified the inhibition of the M6-Fe sample.

### 3.7. TGA Measurement

**Figure 11:**
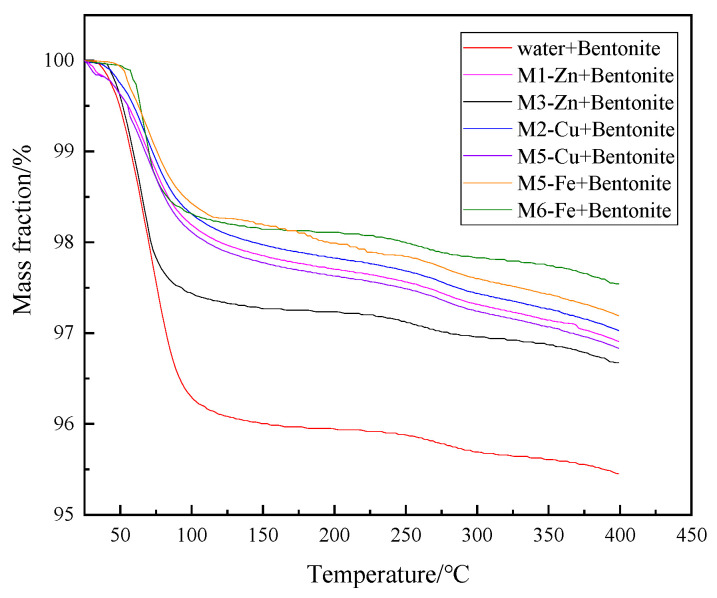
TGA curves of bentonite after different sample treatments.

The TGA curves of the bentonite particles after partial samples and water treatment are shown in Figure 11.

The weight retention of bentonite after treatment with some of the 4% concentration samples remained stable after 100 °C, indicating that the main loss from the bentonite was water. Following research and analysis, we can conclude that this was due to the positive charge of the M-MMH sample opposite the negative charge on the bentonite surface and the lamellar structure of the bentonite being drawn closer under the action of the two different charges, which reduced the interlayer distance of the bentonite and thus inhibited the water absorption of bentonite. As can be seen in Figure 11, M6-Fe-treated bentonite had the smallest loss rate and the best effect of inhibiting bentonite water absorption, better than the other five samples, and M1-Zn had the worst effect of inhibiting bentonite but was still better than the samples treated with water. The water content after treatment with the 4% M6-Fe sample indicated that the M6-Fe sample could strongly inhibit water from entering the interlayer of bentonite and proved that the samples have the ability to inhibit the hydration expansion and hydration dispersion of bentonite.

### 3.8. SEM Observations

**Figure 12:**
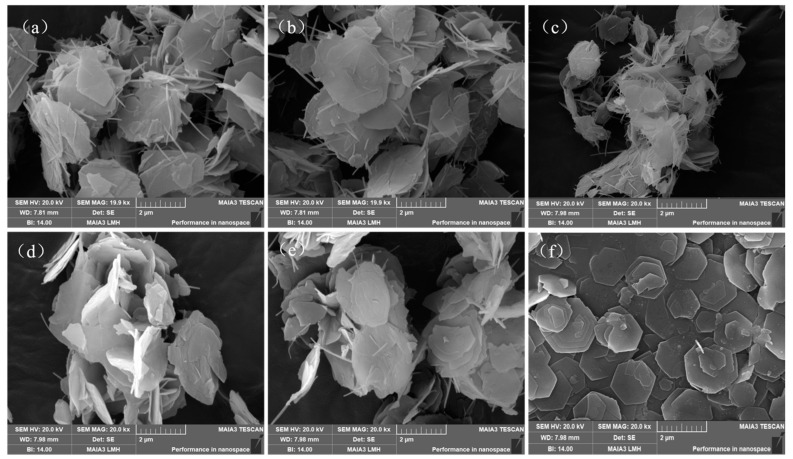
SEM images ((**a**): M1-Zn; (**b**): M3-Zn; (**c**): M2-Cu; (**d**): M5-Cu; (**e**): M5-Fe; (**f**): M6-Fe).

The SEM images of the samples are shown in Figure 12a–f and represent M1-Zn, M3-Zn, M2-Cu, M5-Cu, M5-Fe and M6-Fe, respectively. M6-Fe has the best ortho-hexagonal structure with the most complete and tightly arranged lamellar structure. The other samples have ortho-hexagonal structures, but the arrangements are scattered and the hexagonal structure is incomplete. This proves that the structure formed at Mg:Al:Fe = 3:1:1 (M6-Fe) was the most complete and had the most effective performance.

### 3.9. TEM Observations

**Figure 13:**
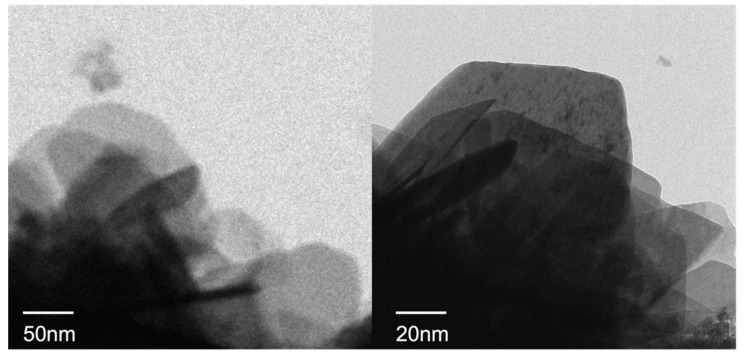
TEM image of M6-Fe.

TEM images of M6-Fe are shown in Figure 13. As can be seen in Figure 13, M6-Fe has a distinct ortho-hexagonal structure. It has been proven that the most complete structure of the sample is formed when Mg:Al:Fe = 3:1:1. From the figure, it can be seen that the sample has a clear layered structure and that the particle size is distributed in the range of 50–150 nm, such that it has the characteristics of a nanomaterial.

### 3.10. Performance in Drilling Fluid

Due to the positive hexagonal lamellar structure of M-MMH, it has a strong temperature resistance while also having viscosity. Viscosity at 600 r/min and 300 r/min after hot rolling at room temperature and 200 °C for 16 h was used to calculate its apparent viscosity (AV/mPa·s), plastic viscosity (PV/mPa·s), yield point (YP/Pa), YP/PV ratio, FL and lubricity factor. The results are shown in Table 3 and Table 4, below.

As can be seen in Table 3, the prepared samples exhibited low viscosity, high yield points and high YP/PV ratios at room temperature. Among all the samples, the M6-Fe drilling fluid base mud exhibited a high YP/PV ratio of 3.23. However, as a drilling fluid base mud, it had too high a rate of filtration loss. The lubrication performance was also bad.

All the formulated M-MMH drilling fluid base mud samples showed decreases in YP/PV ratios after 16h of hot rolling, but the changes were not significant, indicating that stable performance was still maintained at high temperatures. The M6-Fe drilling fluid base mud maintained a high YP/PV ratio of 2.33 after 16 h of hot rolling, which indicated that the M6-Fe drilling fluid base mud had high temperature resistance and low viscosity.

### 3.11. Mechanism

M-MMH has a strong ability to inhibit the hydration expansion and dispersion of bentonite. Meanwhile, M-MMH has temperature resistance, which can improve the yield point of drilling fluid. M-MMH has a hydrotalcite-like structure. During the formation process, part of the Mg^2+^ in brucite is replaced by Al^3+^ isomorphs; the crystal structure does not change and a magnesium–aluminum hydroxide octahedral structure layer is formed, which is hydrotalcite-like and comprises a crystal layer unit of hydrotalcite [30]. Hydrotalcite is a kind of one-sided, brucite-like, surface-overlapping structure, as shown in Figure 13.

**Figure 14:**
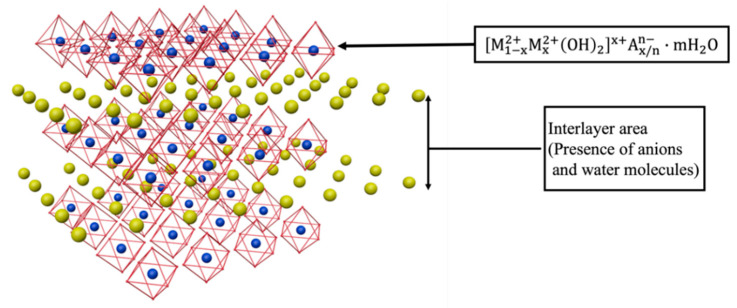
Crystal structure of M-MMH.

**Figure 15:**
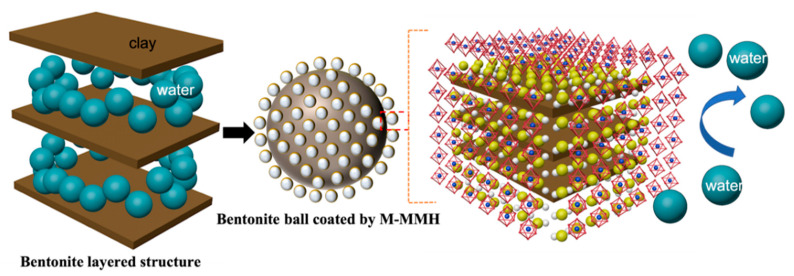
M-MMH inhibition mechanism.

Bentonite consists of silicon–oxygen tetrahedra and aluminum–oxygen octahedra. Bentonite undergoes lattice substitution in water. Si^4+^ is replaced by Al^3+^ in silica–oxygen tetrahedra, and Al^3+^ is replaced by Mg^2+^ in aluminum–oxygen octahedra. This results in the bentonite being negatively charged in water [31]. M-MMH is positively charged in water. Therefore, the bentonite surface will adhere to a layer of M-MMH due to electrostatic adsorption, as shown in Figure 14. This leads to the inability of water molecules to enter between the bentonite layers, thus reducing the expansion of the bentonite (Figure 15).

## 4. Conclusions

The experiments showed that the inhibition of bentonite hydration by M-MMH gradually increased with the increase in the concentrations of samples, among which 4% M6-Fe (Mg:Al:Fe = 3:1:1) had the best inhibition effect. The temperature-resistance experiments showed that M-MMH also had strong inhibition at high temperatures. The M-MMH samples were evaluated as base muds for a drilling fluid test, and it was found that M-MMH has the characteristics of low viscosity and a high yield point; its viscosity and YP/PV ratio did not change much after hot rolling at 200 °C for 16 h, showing that it has strong thermal stability. The linear expansion rate of 4% M6-Fe at 90 °C was 20.28%, with an increase of 70.95% compared to water and of 56.02% compared to the 4% KCl solution. M6-Fe showed the best inhibition, with an 81.16% anti-swelling rate. The above experiments indicated that the M6-Fe sample could strongly inhibit water from entering into the interlayer of bentonite and proved that the sample has the ability to inhibit the hydration expansion and hydration dispersion of bentonite. The M6-Fe drilling fluid base mud maintained a high YP/PV ratio of 2.33 after 16 h of hot rolling, which indicated that the M6-Fe drilling fluid base mud had high temperature resistance and low viscosity. As an inhibitor, M-MMH can effectively inhibit water from entering the well wall and reduce the risk of collapse of the well wall. As a solid-free drilling fluid base mud, M-MMH can increase drilling speed, extend bit life and reduce production cycle time. However, as a drilling fluid base mud, M-MMH has the disadvantages of high filtration loss and poor dispersibility. It is hoped that a complete drilling fluid system can be formed through subsequent research.

## Figures and Tables

**Table 1:** M-MMH molar ratio and names of MMH.

Name	Mg:Al:Zn	Name	Mg:Al:Cu	Name	Mg:Al:Fe
M1-Zn	1:1:1	M1-Cu	1:1:1	M1-Fe	1:1:1
M2-Zn	1:2:1	M2-Cu	1:2:1	M2-Fe	1:2:1
M3-Zn	1:3:1	M3-Cu	1:3:1	M3-Fe	1:3:1
M4-Zn	2:2:1	M4-Cu	2:2:1	M4-Fe	2:2:1
M5-Zn	3:1:0.5	M5-Cu	3:1:0.5	M5-Fe	3:1:0.5
M6-Zn	3:1:1	M6-Cu	3:1:1	M6-Fe	3:1:1
M7-Zn	3:1:2	M7-Cu	3:1:2	M7-Fe	3:1:2
M8-Zn	3:1:3	M8-Cu	3:1:3	M8-Fe	3:1:3

**Table 2:** Average and median particle sizes of bentonite under different treatments.

Name	Average Particle Size/μm	Median Particle Size/μm
Bentonite	36.42 (±3.1)	32.18 (±0.6)
Bentonite + water	17.12 (±1.7)	12.63 (±0.5)
Bentonite + M1-Zn	26.74 (±2.4)	21.16 (±1.1)
Bentonite + M4-Zn	27.75 (±2.1)	24.54 (±1.1)
Bentonite + M2-Cu	32.86 (±3.2)	26.59 (±0.7)
Bentonite + M5-Cu	30.76 (±3.4)	29.62 (±0.6)
Bentonite + M5-Fe	36.12 (±2.8)	31.17 (±1.2)
Bentonite + M6-Fe	38.75 (±3.3)	33.45 (±1.0)

**Table 3:** Rheological properties of the 4% samples at room temperature.

Name	AV/mPa·s	PV/mPa·s	YP/Pa	YP/PV	FL/mL	Lubricity Factor
M1-Zn	2.75 (±0.32)	1.50 (±0.04)	1.25 (±0.01)	0.83 (±0.02)	113.2 (±10.4)	0.14 (±0.01)
M2-Zn	3.25 (±0.12)	1.50 (±0.03)	1.75 (±0.02)	1.17 (±0.04)	124.5 (±16.2)	0.12 (±0.01)
M3-Zn	3.25 (±0.21)	1.60 (±0.04)	1.65 (±0.02)	1.03 (±0.03)	108.6 (±9.6)	0.14 (±0.01)
M4-Zn	2.25 (±0.17)	1.00 (±0.07)	1.25 (±0.04)	1.25 (±0.04)	156.2 (±22.0)	0.18 (±0.02)
M5-Zn	4.00 (±0.30)	1.50 (±0.02)	2.50 (±0.03)	1.67 (±0.03)	144.8 (±10.4)	0.18 (±0.01)
M6-Zn	3.75 (±0.18)	1.50 (±0.05)	2.25 (±0.05)	1.50 (±0.05)	131.4 (±16.4)	0.13 (±0.02)
M7-Zn	2.50 (±0.09)	1.00 (±0.07)	1.50 (±0.03)	1.50 (±0.03)	144.2 (±18.0)	0.11 (±0.01)
M8-Zn	2.25 (±0.11)	1.00 (±0.10)	1.25 (±0.06)	1.25 (±0.06)	110.4 (±16.8)	0.11 (±0.01)
M1-Cu	2.00 (±0.24)	1.50 (±0.06)	0.50 (±0.05)	0.33 (±0.04)	122.8 (±17.2)	0.05 (±0.01)
M2-Cu	2.50 (±0.22)	1.00 (±0.04)	1.50 (±0.01)	1.50 (±0.02)	130.0 (±12.8)	0.18 (±0.01)
M3-Cu	3.00 (±0.13)	2.00 (±0.03)	1.00 (±0.06)	0.50 (±0.01)	117.2 (±21.4)	0.19 (±0.02)
M4-Cu	2.25 (±0.24)	1.50 (±0.08)	0.75 (±0.02)	0.50 (±0.02)	142.2 (±19.8)	0.19 (±0.01)
M5-Cu	4.25 (±0.31)	2.50 (±0.03)	1.75 (±0.08)	0.70 (±0.06)	102.2 (±16.4)	0.11 (±0.01)
M6-Cu	2.25 (±0.23)	1.00 (±0.01)	1.25 (±0.06)	1.25 (±0.04)	134.2 (±10.2)	0.11 (±0.01)
M7-Cu	5.00 (±0.41)	1.50 (±0.01)	3.50 (±0.06)	2.33 (±0.11)	110.6 (±11.0)	0.13 (±0.01)
M8-Cu	3.75 (±0.33)	1.50 (±0.01)	2.25 (±0.04)	1.50 (±0.10)	132.0 (±17.8)	0.27 (±0.01)
M1-Fe	2.00 (±0.15)	1.50 (±0.02)	0.50 (±0.01)	0.33 (±0.01)	156.6 (±10.4)	0.28 (±0.01)
M2-Fe	1.75 (±0.17)	1.00 (±0.04)	0.75 (±0.03)	0.75 (±0.04)	118.8 (±20.8)	0.29 (±0.02)
M3-Fe	2.25 (±0.20)	1.00 (±0.07)	1.25 (±0.02)	1.25 (±0.02)	108.2 (±16.6)	0.23 (±0.01)
M4-Fe	1.50 (±0.10)	1.00 (±0.05)	0.50 (±0.03)	0.50 (±0.07)	114.0 (±21.4)	0.21 (±0.01)
M5-Fe	4.50 (±0.47)	1.50 (±0.05)	3.00 (±0.08)	2.00 (±0.12)	126.8 (±19.2)	0.20 (±0.01)
M6-Fe	5.50 (±0.22)	1.30 (±0.02)	4.20 (±0.11)	3.23 (±0.14)	105.6 (±12.0)	0.17 (±0.01)
M7-Fe	4.00 (±0.41)	1.50 (±0.07)	2.50 (±0.16)	1.67 (±0.04)	116.8 (±11.4)	0.25 (±0.01)
M8-Fe	4.50 (±0.51)	2.00 (±0.05)	2.50 (±0.03)	1.25 (±0.06)	134.8 (±8.8)	0.24 (±0.01)

**Table 4:** Rheological properties of the 4% samples after 16 h of hot rolling at 200 °C.

h	AV/mPa·s	PV/mPa·s	YP/Pa	YP/PV	FL/mL	Lubricity Factor
M1-Zn	1.50 (±0.12)	1.00 (±0.10)	0.50 (±0.05)	0.50 (±0.03)	124.2 (±11.4)	0.13 (±0.01)
M2-Zn	2.00 (±0.10)	1.00 (±0.08)	1.00 (±0.03)	1.00 (±0.07)	133.5 (±14.2)	0.11 (±0.01)
M3-Zn	2.50 (±0.08)	1.50 (±0.12)	1.00 (±0.11)	0.67 (±0.08)	123.6 (±11.6)	0.15 (±0.01)
M4-Zn	1.50 (±0.11)	1.00 (±0.11)	0.50 (±0.03)	0.50 (±0.11)	176.2 (±19.0)	0.13 (±0.02)
M5-Zn	2.50 (±0.09)	1.00 (±0.05)	1.50 (±0.03)	1.50 (±0.12)	175.8 (±17.4)	0.15 (±0.01)
M6-Zn	3.00 (±0.21)	1.50 (±0.07)	1.50 (±0.02)	1.00 (±0.03)	145.4 (±19.4)	0.17 (±0.02)
M7-Zn	3.25 (±0.22)	1.50 (±0.09)	1.75 (±0.11)	1.17 (±0.05)	134.2 (±28.0)	0.18 (±0.01)
M8-Zn	2.75 (±0.17)	1.50 (±0.11)	1.25 (±0.15)	0.83 (±0.08)	124.4 (±6.8)	0.17 (±0.01)
M1-Cu	3.00 (±0.18)	2.00 (±0.14)	1.00 (±0.03)	0.50 (±0.09)	134.8 (±13.2)	0.08 (±0.01)
M2-Cu	2.50 (±0.15)	2.00 (±0.05)	0.50 (±0.05)	0.25 (±0.01)	124.0 (±18.8)	0.12 (±0.01)
M3-Cu	3.00 (±0.11)	2.00 (±0.15)	1.00 (±0.07)	0.50 (±0.05)	152.2 (±21.4)	0.14 (±0.02)
M4-Cu	2.50 (±0.25)	2.00 (±0.04)	0.50 (±0.10)	0.25 (±0.02)	161.2 (±19.8)	0.18 (±0.01)
M5-Cu	2.00 (±0.19)	1.50 (±0.05)	0.50 (±0.02)	0.33 (±0.03)	114.2 (±16.4)	0.13 (±0.01)
M6-Cu	2.25 (±0.18)	1.50 (±0.13)	0.75 (±0.07)	0.50 (±0.03)	146.2 (±10.2)	0.17 (±0.01)
M7-Cu	2.50 (±0.24)	1.00 (±0.21)	1.50 (±0.08)	1.50 (±0.02)	131.6 (±31.0)	0.12 (±0.01)
M8-Cu	3.00 (±0.32)	1.50 (±0.15)	1.50 (±0.07)	1.00 (±0.04)	139.0 (±27.8)	0.24 (±0.01)
M1-Fe	3.00 (±0.17)	2.50 (±0.13)	0.50 (±0.03)	0.20 (±0.01)	134.6 (±10.4)	0.26 (±0.01)
M2-Fe	1.50 (±0.16)	1.00 (±0.17)	0.50 (±0.02)	0.50 (±0.02)	146.8 (±23.8)	0.23 (±0.02)
M3-Fe	3.00 (±0.18)	1.00 (±0.12)	2.00 (±0.11)	2.00 (±0.11)	125.2 (±15.6)	0.23 (±0.01)
M4-Fe	1.75 (±0.07)	1.50 (±0.09)	0.25 (±0.03)	0.17 (±0.01)	145.0 (±25.4)	0.21 (±0.01)
M5-Fe	2.00 (±0.21)	1.30 (±0.14)	0.70 (±0.04)	0.54 (±0.02)	134.8 (±18.2)	0.20 (±0.01)
M6-Fe	5.00 (±0.31)	1.50 (±0.16)	3.50 (±0.23)	2.33 (±0.13)	111.6 (±14.0)	0.17 (±0.01)
M7-Fe	2.50 (±0.12)	1.50 (±0.10)	1.00 (±0.12)	0.67 (±0.07)	131.8 (±14.4)	0.25 (±0.01)
M8-Fe	2.00 (±0.22)	1.50 (±0.09)	0.50 (±0.05)	0.33 (±0.03)	148.8 (±18.8)	0.24 (±0.01)
